# Female Wild Turkeys Exhibit Context Dependence in Their Selection of Roads

**DOI:** 10.1002/ece3.74468

**Published:** 2026-10-14

**Authors:** Kyle J. Smelter, Erik J. Blomberg, Liam A. Berigan, Matthew B. Gonnerman, Stephanie A. Shea, Veronica A. Winter, Kelsey Sullivan, Pauline L. Kamath, Frances E. Buderman

**Affiliations:** ^1^ Department of Ecosystem Science and Management The Pennsylvania State University University Park Pennsylvania USA; ^2^ Department of Wildlife, Fisheries, and Conservation Biology The University of Maine Orono Maine USA; ^3^ College of Forestry, Wildlife and Environment Auburn University Auburn Alabama USA; ^4^ Department of Environmental Science and Technology University of Maryland College Park Maryland USA; ^5^ Post‐doctoral Associate with the U.S. Geological Survey Eastern Ecological Science Center Laurel Maryland USA; ^6^ Cooperative Extension The University of Maine Orono Maine USA; ^7^ Maine Department of Inland Fisheries and Wildlife Augusta Maine USA; ^8^ School of Food and Agriculture The University of Maine Orono Maine USA

**Keywords:** habitat selection, *Meleagris gallopavo*, movement ecology, road ecology, season, telemetry

## Abstract

It is essential for wildlife managers to understand how animals interact with and respond to the landscape. However, wildlife‐habitat relationships are often context‐dependent, with habitat selection influenced by abiotic and biotic factors. Recognizing this, we investigated how roads influenced female wild turkey ( *Meleagris gallopavo silvestris* ; hereafter female) seasonal habitat selection in Maine, USA, as a function of road type, time‐of‐day, and land cover type to identify context‐dependent responses. Because previous research has demonstrated that wildlife can display variable selection patterns in response to different road types, we assessed differences in selection between primary roads (state roads and highways) and secondary roads (local/township roads). When modeling baseline differences in seasonal habitat selection patterns, we found that females selected areas closer to secondary roads during winter and late spring and selected areas farther from primary roads during long‐distance movements in early spring. When modeling differences in selection of roads across time‐of‐day and season, we found that females selected locations closer to secondary roads during winter and summer mornings, periods typically associated with elevated traffic. When modeling the effect of land cover type on seasonal habitat selection relative to hardwood forest, we found that females selected locations farther from primary and secondary roads in developed landscapes across most seasons. However, relative to hardwood forest, females selected locations closer to primary roads near conifer forest cover during winter. Notably, we observed considerable individual variation in seasonal selection of primary and secondary roads, demonstrating heterogeneity in selection patterns between individuals within the study. Our findings have implications for understanding the temporal risk of wildlife‐vehicle collisions and suggest that factors such as season, time‐of‐day, and the surrounding landscape context of roads influence habitat selection, warranting consideration when evaluating the ecological effects of roads.

## Graphical abstract

We investigated how roads influenced female wild turkey ( *Meleagris gallopavo silvestris* ; hereafter female) seasonal habitat selection in Maine USA as a function of road type, time of day, and land cover type to discern how females may select for areas near roads based on different contexts. We found female relationships with roads to be highly context‐dependent, with individuals displaying variability in selection patterns such as selection for areas near secondary roads particularly in the morning hours in some seasons and selection for areas farther from roads in developed landscapes. Our findings have implications for understanding the temporal risk of wildlife‐vehicle collisions and suggest that factors such as season, time‐of‐day, and the surrounding landscape context of roads influence habitat selection, warranting consideration when evaluating the ecological effects of roads.

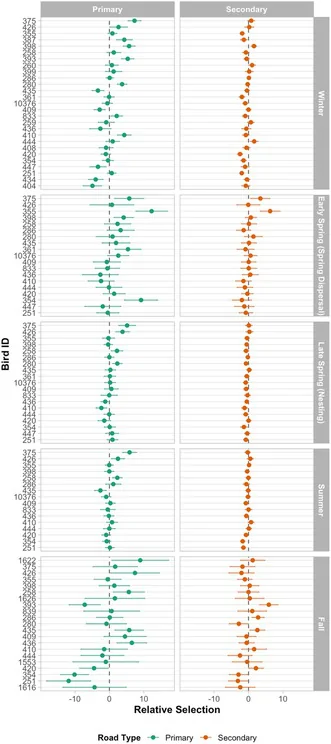

## 1 Introduction

Roads are a dominant feature of modern landscapes, shaping the movement of humans and the distribution of global resources (Davenport and Davenport 2006). In addition, roads may influence the behavior, distribution, and survival of wildlife (Forman and Alexander 1998; Coffin 2007). For example, roads are a primary cause of landscape fragmentation, which can impede population connectivity and reduce habitat quality for many, but not all, wildlife species (Mader 1984; Saunders et al. 1991; Shepard et al. 2008). In addition, roads contribute to mortality risk through wildlife‐vehicle collisions and may cause stress due to anthropogenic disturbance, such as vehicle noise (Forman and Alexander 1998; Spellerberg 1998). Conversely, roads may serve as travel corridors for some species, allowing individuals to access and exploit otherwise disconnected resources (Latham et al. 2011; Raiter et al. 2018; Hill et al. 2021). As a result, the influence of roads on wildlife may be highly context‐dependent and vary according to the species' ecology, environmental factors, and individual variables (Clevenger et al. 2015). Due to this, a better understanding of how wildlife interacts with roads will benefit species conservation and management.

Given the widespread distribution of wild turkeys ( *Meleagris gallopavo silvestris* ; hereafter turkeys) across both rural and developed landscapes, understanding how individuals respond to roads has important implications for conservation and management. Previous studies suggest that turkeys may respond to roads differently across seasons, times of day, and levels of anthropogenic disturbance (Butler et al. 2005; Erxleben et al. 2011; Carl et al. 2023). For example, Nelson et al. (2023) reported that turkeys in Georgia, USA selected areas closer to trails and low‐use forest roads during brood‐rearing, which was posited to reflect exploitation of food resources near habitat edges. In addition, McDougal et al. (1990) found that turkeys in Virginia tended to avoid high traffic roads, only crossing near forested sections, likely due to increased concealment and perceived safety. Road proximity has been negatively linked to turkey survival, due to ease of access for hunters (Holbrook and Vaughan 1985; Thogmartin and Schaeffer 2000; Gerrits et al. 2020) and wildlife‐vehicle collisions. Previous studies have found that turkey occupancy may be negatively associated with roads in developed areas, suggesting that female turkeys (hereafter females) may balance space use with the threat of mortality (Fleming and Porter 2000; Collins et al. 2025). However, Gulotta et al. (2026) found that turkey fitness may be greater in urban areas displaying evidence for the human shield hypothesis and suggesting future research is needed to fully disentangle this relationship. As such, understanding how females interact with roads under varying environmental and temporal conditions can provide insights into behavioral adaptations in human‐modified landscapes.

In this study, we explored how road selection of females varied with season, time‐of‐day, and land cover type. To further discern differences in selection between different road types, we analyzed selection of primary roads (state roads and highways) and secondary roads (local roads) independently. Our objectives were to (1) evaluate seasonal variation in primary and secondary road selection, (2) assess how primary and secondary road selection varied across times of day within each season, and (3) determine how the surrounding landscape influenced primary and secondary road selection across different seasons. To meet our objectives, we analyzed a dataset of female turkey movements in central Maine, USA, testing a series of hypotheses and associated predictions anticipating that road selection would be highly context‐dependent. We hypothesized that the surrounding landscape would largely influence road selection. We predicted that females in developed landscapes would select areas farther from secondary roads to reduce exposure to predators that may use road edges as travel corridors. We hypothesized that time‐of‐day would influence seasonal patterns of road selection. We predicted that females would select locations closer to secondary roads in the morning hours as they often move from roost sites to nearby foraging areas (Huang et al. 2026) and roadside edges may provide accessible forage and efficient travel corridors. In addition, we predicted that females would select areas farther from secondary roads at midday, as they have already made their foraging movements from the roost. We hypothesized that selection would be influenced by the type of road encountered. We predicted that females would select locations farther from primary roads in all land cover types due to greater vehicle noise and perceived collision risk.

## 2 Materials and Methods

### 2.1 Study Area

The study area consisted of five wildlife management districts (WMDs) in central Maine, USA: 14, 17, 18, 23, and 26 each with varying land cover compositions (Figure 1; Table 1). Urban development and human population density were greatest in WMD 26 and lowest in WMD 14. Maine has a strong seasonal climate characterized by cold winters with snow accumulation and warm summers. During the study period, the mean daily temperature was −6.32°C in January and 20.37°C in July (National Centers for Environmental Information). Mean daily precipitation was 11.07 cm in January occurring as primarily snowfall and 9.50 cm in July occurring primarily as rainfall (National Centers for Environmental Information). Dominant forest types in the study area were spruce‐fir and northern hardwood forests, with both large‐scale commercial ownership as well as smaller privately owned forest parcels. Common softwood tree species in the study area consisted of red spruce ( *Picea rubens* ), balsam fir ( *Abies balsamea* ), eastern white pine ( *Pinus strobus* ), and eastern hemlock ( *Tsuga canadensis* ) (Butler 2018). Common hardwood trees included red maple ( *Acer rubrum* ), sugar maple ( *Acer saccharum* ), paper birch (*Betula popula*), and yellow birch ( *Betula alleghaniensis* ) (Butler 2018).

**FIGURE 1:**
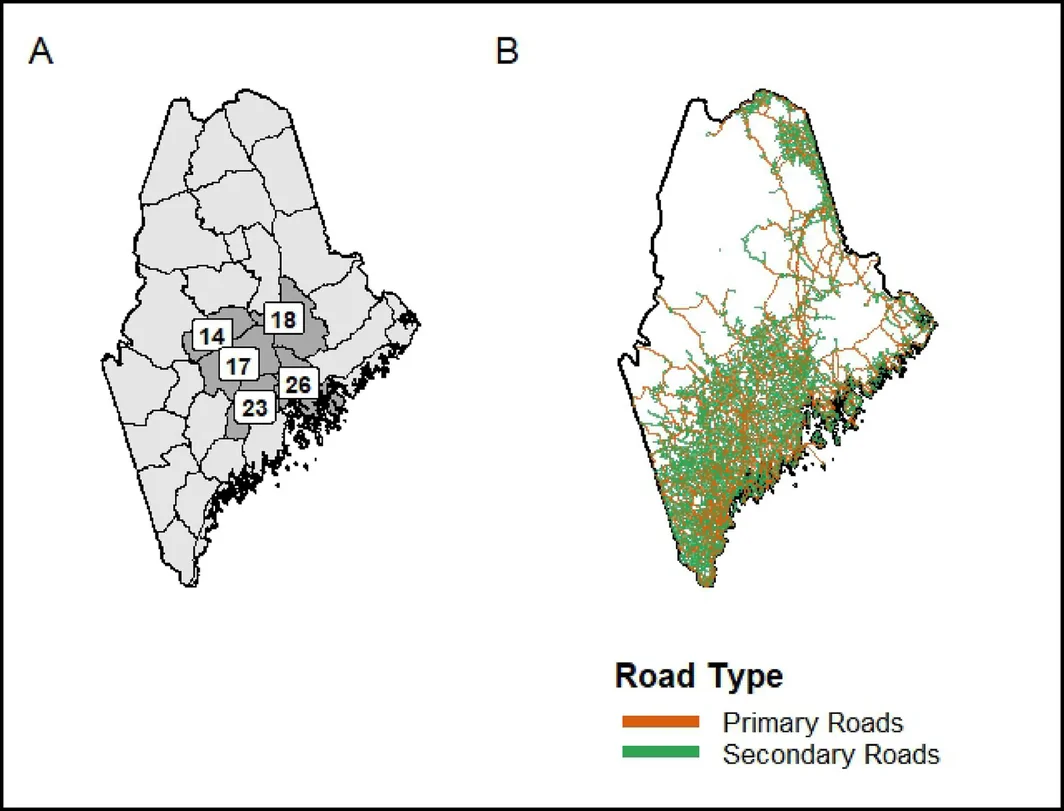
The study area spanned five wildlife management districts (WMDs) including 14, 17, 18, 23, and 26 in central Maine, USA (2018–2020; A). Primary (state roads and highways; brown lines; B) and secondary roads (local and township roads; green lines; B) were distributed throughout the study area. 1–11 female eastern wild turkeys ( *Meleagris gallopavo silvestris* ) were captured and fitted with a GPS radio transmitter in each WMD.

**TABLE 1:** Percent land cover type in Wildlife Management Districts 14, 17, 18, 23, and 26, in central Maine, USA (2018–2020).

WMD	% Developed	% Hardwood	% Conifer	% Agriculture	% Other
14	2	24	59	< 1	12
17	5	31	47	9	6
18	3	31	55	1	8
23	9	35	40	9	7
26	10	22	51	3	12

### 2.2 Field Methods

#### 2.2.1 Turkey Capture and Processing

We captured females at sites baited with cracked corn during three winters (2018–2020) using both rocket (Grubb 1988) and drop nets (Glazener et al. 1964). We equipped a subset of females with 90‐g Lotek GPS transmitters (Lotek Wireless, Newmarket, Ontario, CA) attached via a backpack‐style harness. Transmitters did not exceed 4% of the bird's body mass. The GPS units were programmed to record hourly locations during daylight hours from 1 November through 31 July, and a single roost location each night at either 12:00 a.m. or 1:00 a.m. (Cohen et al. 2018). To conserve transmitter battery life for multiple nesting seasons which were needed for other study objectives, GPS units were programmed to collect two locations during the daylight hours and a single roost observation between 1 August and 31 October. Location data were downloaded weekly using a handheld antenna and receiver (Pinpoint Commander, Lotek Wireless, Newmarket, Ontario, CA). We censored all individuals that died within 2 weeks of capture to account for trapping‐related effects on movement. Although several females within a flock could be tagged and monitored, we used GPS data from one randomly selected female per captured flock in our analysis to minimize pseudo‐replication (see Gonnerman et al. 2023). For females flocking together, we selected data for a single bird as input to the model. If only one individual in a flock survived the winter, this bird was used as an input to maximize data. If more than one bird survived the winter, we randomly selected an individual from those remaining. All animal handling protocols were approved by the University of Maine's Institutional Animal Care and Use Committee (IACUC Protocol # A2017_11_03).

### 2.3 Data Management

#### 2.3.1 Road Categorization

We obtained public road location data from the Maine Department of Transportation via the Maine Office of GIS (Kane 2019) and categorized roads into two groups: primary roads, which included state roads, arterials, collectors, and highways; and secondary roads, which consisted of local (i.e., municipal or township) roads (Table 2). Using the *sf* (Pebesma and Bivand 2023) and *terra* (Hijmans 2026) R packages, we calculated the Euclidean distance from each GPS location to the nearest primary and secondary road, which we summarized as separate raster layers with a 30 m resolution.

**TABLE 2:** Road delineation used to evaluate the influence of roads on habitat selection by female eastern wild turkeys ( *Meleagris gallopavo silvestris* ) in central Maine, USA, from 2018 to 2020.

Road classification	Description	Defined road type
Interstate	Connect to state highways and arterials	Primary
Major collector	Collectors link smaller towns, villages, neighborhoods	Primary
Minor collector	*See major collector	Primary
Minor arterial	Arterials serve countywide, statewide or interstate travel	Primary
Other freeway or expressway	Expressways connect local roads to the state highway system	Primary
Other principal arterial	*See minor arterial	Primary
Local	Provide direct access to residential neighborhoods, local businesses etc.	Secondary

#### 2.3.2 Defining Seasons and Time‐of‐Day

To examine seasonal differences in road selection, we defined seasonal occurrence distributions using dynamic Brownian bridge movement models (dBBMMs; Kranstauber et al. 2012), which we generated at the individual level to account for variation in movement phenology. Following Gonnerman et al. (2022), we fit dBBMMs to sequences of GPS locations for each individual and extracted estimates of Brownian motion variance (*σ* ^2^_*m*_), which reflects the relative speed and tortuosity of an individual's movements (Byrne et al. 2014). We calculated daily *σ* ^2^_*m*_ estimates by averaging all *σ* ^2^_*m*_ values within a day and plotted daily averages as a time series for each individual. Time series plots were visually assessed to identify sustained shifts in movement behavior including abrupt increases, decreases, and changes in mean variance. These shifts were used to delineate seasonal date boundaries for each individual that aligned with a priori seasons within the turkey annual cycle, including winter, early spring (spring dispersal), late spring (nesting), summer, and fall. Winter was characterized by consistently low daily *σ* ^2^_*m*_ values. Early spring (spring dispersal) was identified by steep increases in daily *σ* ^2^_*m*_ indicative of long‐distance directional movements without recursion. Not all individuals exhibited a defined spring dispersal movement; thus, analyses for early spring were restricted to individuals that underwent movements. Following a spring dispersal movement, nesting was characterized by a return to moderate daily *σ* ^2^_*m*_ levels. The nesting category contained all aspects of female movements associated with selecting a nest site following dispersal, including pre‐nesting movements that were presumably associated with nest site selection and movements during the laying stage before females began incubation. We removed GPS locations during incubation to exclude periods of stationary behavior and repeated sampling of the nest location from analyses. Summer was defined as moderate to high *σ* ^2^_*m*_ with variability among individuals likely reflecting nest success and whether females exhibit brood‐rearing behavior. We used an a priori estimate for the fall season (1 August–31 October), which aligned with observed changes in turkey behavior and our interest in assessing how road selection differed during Maine's fall turkey harvest season, which included opportunities to pursue females. All GPS locations were assigned to seasons based on these individual‐specific classifications.

We classified time‐of‐day into three categories according to a priori assumptions of diurnal turkey behavioral states and used the *suncalc* package (Thieurmel and Elmarhraoui 2026) to estimate daily sunrise and sunset times. We first estimated the solar midpoint for each day (sunrise + (sunset‐sunrise)/2) and created a midday category by subtracting 2 h from the solar midpoint and then adding 2 h to the solar midpoint to create a 4‐h window. We assumed that midday encapsulated loafing behavior. Morning consisted of times between daily sunrise and the start of the midday window, which we assumed encapsulated movement from roost sites and foraging behavior. Afternoon consisted of times between the end of the midday window and daily sunset, which we assumed consisted of late‐day movements and roost selection. We matched GPS locations to each time‐of‐day category to assess how road selection differed throughout the day. We removed GPS locations after sunset and before sunrise as that characterized stationary roosting behavior.

#### 2.3.3 Land Cover Categorization

We processed spatial land cover data from the 2019 National Land Cover Database (NLCD; Dewitz 2021), and used the *tigris* (Walker 2025) and *FedData* (Bocinsky 2024) R packages to assess the relationship of landscape variables with road selection. The NLCD portrays land cover information as grid cells with a 30 m × 30 m spatial resolution. We grouped NLCD classifications into four categories that accounted for different land cover compositions available to females (Table 3). To represent different forested landscapes, a Hardwood category was formed by combining deciduous and wooded wetland cover, and a Conifer category was created by merging evergreen and mixed forest cover. To represent human‐developed areas, we merged the categories for developed space, low‐intensity developed, medium‐intensity developed, and high‐intensity developed into a Developed category. We created the single Developed category due to a low sample size of females in each level of urban development within the study area. Finally, an Agriculture category was created by merging pasture/hay and cultivated crop cells to determine if female selection of roads varied in agricultural landscapes.

**TABLE 3:** Land cover variables derived from the 2021 National Land Cover Database used to model female wild turkey ( *Meleagris gallopavo silvestris* ) seasonal road selection in Central Maine USA (2018–2020).

Land cover variable	Definition	References
Hardwood	Deciduous forest cells	Niedzielski and Bowman (2016)
Conifer	Evergreen and mixed forest cells	Gonnerman et al. (2023)
Developed	Low, medium, and high intensity developed cells	Gulotta et al. (2026) Collins et al. (2025)
Agriculture	Cultivated crop and pasture/hay cells	Vander Haegen et al. (1989)

*Note:* We present each land cover variable included in the analyses, the National Land Cover Database categories that were merged to create the variable, and the supporting literature used to justify their inclusion.

### 2.4 Step‐Selection Functions

We modeled individual selection during each season within our analysis using a step‐selection function (hereafter SSF; Fortin et al. 2005; Thurfjell et al. 2014). We defined our SSF using a conditional Poisson model with stratum‐specific fixed intercepts, which can be estimated by including a random effect with a large, fixed variance (Warton and Shepherd 2010; Muff et al. 2020). Each stratum contained a used location and 10 paired available locations which represented potential alternate movement trajectories that the animal could have taken. Prior to generating available locations, we removed all observations from an individual's movement track that contained a step length value of zero to remove stationary periods. Rather than generating availability from dBBMMs, we constrained available locations according to the observed distribution of step lengths and turning angles from all individuals within our analysis using the *amt* package (Signer et al. 2019). We modeled each used observation (*t*) for individual (*j*) according to a Poisson distribution such that:

(1) $$y_{\mathrm{tj}}\sim \text{Poisson}\left(\mu_{\mathrm{tj}}\right)$$

(2) $$\mu_{\mathrm{nj}}=\exp\left(\alpha_{\mathrm{nt}}+\beta^{\prime }x_{\mathrm{tj}}+u^{\prime }z_{\mathrm{tj}}\right),$$

where $\alpha_{\mathrm{nt}}$ was a stratum‐specific random intercept where the stratum defines the paired used and available locations, $\;\beta^{\prime }$ was a vector of coefficients that describe the effects of covariates $x_{\mathrm{tj}}$, including both main effects and interactions. We also estimated random slopes $z_{\mathrm{tj}}$, for each female to account for individual variation in selection of roads across different contexts. Following Muff et al. (2020), we fixed the variance at 10^6^ to avoid shrinkage when estimating the values of our stratum‐specific random effect. All models were fitted using the *glmmTMB* package (Brooks et al. 2017) in program R (R Core Team 2026). We evaluated uncertainty associated with each estimate using 85% confidence intervals. We inferred selection for areas farther from roads if the estimate and confidence intervals were positive and didn't overlap zero. We inferred selection for areas closer to roads if the estimate and confidence intervals were negative and didn't overlap zero. We did not fit models for the fall season for the time‐of‐day model suite due to zero observations in the midday and afternoon hours.

### 2.5 Statistical Analysis

Given the number of variables and time periods of interest, we fit separate models for each objective (Table 4). For all scenarios, we fit separate models for each road type due to high correlations between distance to primary and secondary roads for used and available locations and therefore could not be included within the same model. First, we fit separate models for each season to gauge baseline selection of each type of road during each period (hereafter season model suite; 10 models). Additionally, we fit models to assess how selection of roads differed by time‐of‐day and season (hereafter time‐of‐day model suite; 24 models). We subset the data by the appropriate period of interest (e.g., winter morning) and fit models containing only a main effect of distance to road type for each period (Table 3). Lastly, we fit models to gauge how surrounding land cover context influenced the selection of roads by season and land cover type (hereafter landscape model suite; 10 models).

**TABLE 4:** Models and data partitions used to address each study objective evaluating the effects of season, time‐of‐day, and land cover type on female eastern wild turkey ( *Meleagris gallopavo silvestris* ) seasonal road selection in central Maine USA.

Objective	Models in suite	Data partition	Main effects	Interaction effects
Objective 1: Seasonal differences in road selection	10	Season	Distance to road by type	None
Objective 2: Effect of time of day on road selection across seasons	24	Season Time of day	Distance to road by type	None
Objective 3: Effect of land cover on road selection across seasons	10	Season	Distance to road by type + land cover	Distance to road by type × land cover

*Note:* The number of separate models within the analysis is presented next to each objective. Depending on the objective, we either partitioned data based on five seasons (winter, early spring, late spring, summer, and fall) or five seasons and three times of day (morning, midday, and afternoon). Road type was categorized as primary or secondary; each model and data partition was fit independently with either distance to primary or secondary road, as these predictors were correlated. Land cover types included hardwood, conifer, developed, and agriculture.

## 3 Results

We used GPS data from 32 females (*n* = 34,688 locations); 12,800 locations were categorized as morning, 11,873 as midday, and 10,015 as afternoon. Data were obtained from five seasonal periods with variability in coverage due to individual Brownian motion variance estimates: winter (4 February–2 May), early spring (11 April–6 May), late spring (16 April–22 June), summer (13 May–31 July), and fall (1 August–31 October). These data consisted of 27 females and 15,820 locations categorized as winter, 19 females and 949 locations as early spring, 19 females and 7858 locations as late spring, 16 females and 9624 locations as summer, and 21 females and 437 locations as fall. The mean hourly step length was 121.56 m in winter, 290.04 m during early spring, 185.38 m during late spring, 146.16 m in summer, and 450.51 m in fall. Landscape use and mean distance to road type varied by season, with conifer forest representing the most frequently used cover type across seasons (Table 5). Selection coefficients and their associated 95% confidence intervals are presented in the Supporting Information files to evaluate uncertainty in effects (Figures S1–S3). We also present the estimated variance from the individual random slope in each model (Tables S1–S3) and the results in the context of relative selection strength (Avgar et al. 2017; Tables S4–S6) in the Supporting Information files.

**TABLE 5:** Variability in seasonal landscape use of female eastern wild turkeys ( *Meleagris gallopavo silvestris* ) in central Maine, USA (2018–2020).

Season	Developed	Hardwood	Conifer	Agriculture	Other
Winter	1801 (0.11)	4361 (0.28)	7319 (0.46)	1454 (0.09)	885 (0.05)
Early spring	190 (0.20)	223 (0.23)	338 (0.36)	139 (0.15)	59 (0.06)
Late spring	1256 (0.16)	1588 (0.20)	2432 (0.31)	1847 (0.24)	735 (0.09)
Summer	1342 (0.14)	1963 (0.20)	4022 (0.42)	1405 (0.15)	892 (0.09)
Fall	106 (0.24)	55 (0.13)	116 (0.27)	104 (0.24)	56 (0.12)

*Note:* For each land cover type and season, the total number of GPS locations is presented above the proportion of locations within that land cover class (*n*) relative to the entire seasonal GPS sample. Calculations were conducted separately for winter (*n* = 27 females), early spring (*n* = 19), late spring (*n* = 19), summer (*n* = 16), and fall (*n* = 21). The land cover proportions do not sum to 100 due to rounding.

### 3.1 Seasonal Differences in Road Selection

Females selected areas closer to secondary roads during winter (−0.48; CI = −0.86 to −0.09) and late spring (−0.48; CI = −0.79 to −0.18; Figure 2). In early spring, summer, and fall, we failed to detect an effect of distance to secondary road on relative selection. When considering distance to primary roads, females selected locations farther from primary roads during early spring (2.15; CI = 0.27–4.04; Figure 2). In addition, when accounting for individual variation in road selection, we observed considerable individual variation in seasonal selection of roads (Figure 3).

**FIGURE 2:**
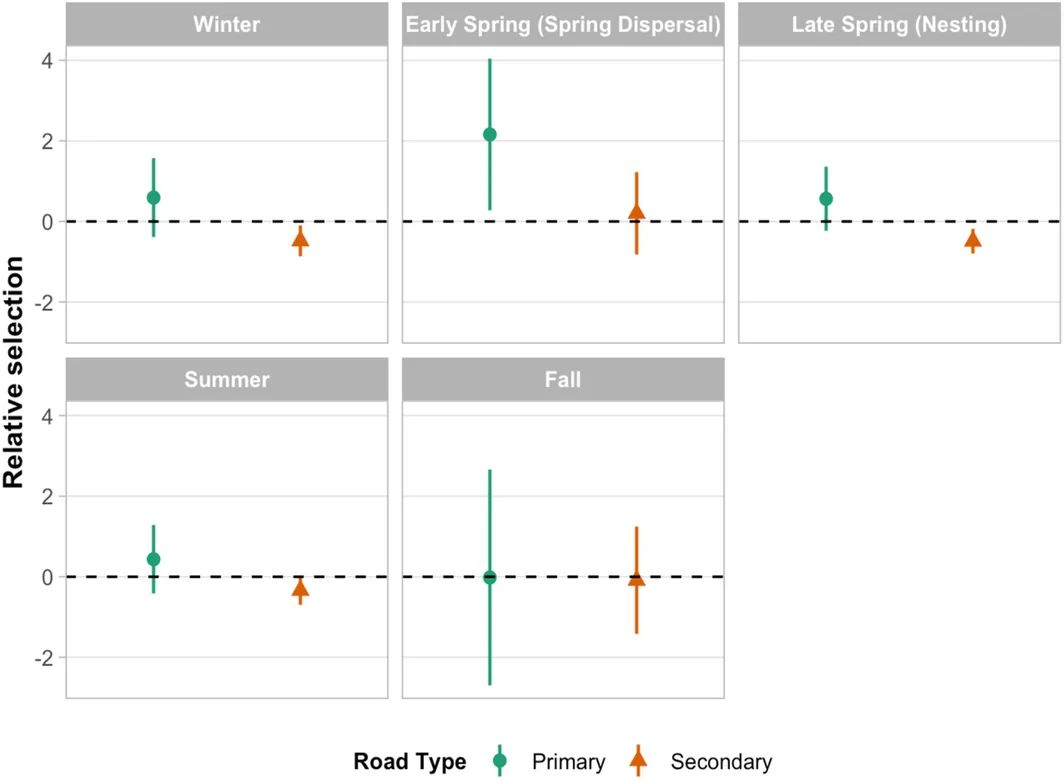
Estimates and associated 85% confidence intervals for mixed effects conditional Poisson regression fit separately for each combination of season modeling female eastern wild turkey ( *Meleagris gallopavo silvestris* ) road selection. Positive coefficients reflect selection for greater distances from roads, while negative coefficients represent selection for areas closer to roads. Habitat selection was modeled separately for winter (*n* = 27 females), early spring (*n* = 19), late spring (*n* = 19), summer (*n* = 16), and fall (*n* = 21). Females were monitored using GPS transmitters in central Maine, USA, from 2018 to 2020.

**FIGURE 3:**
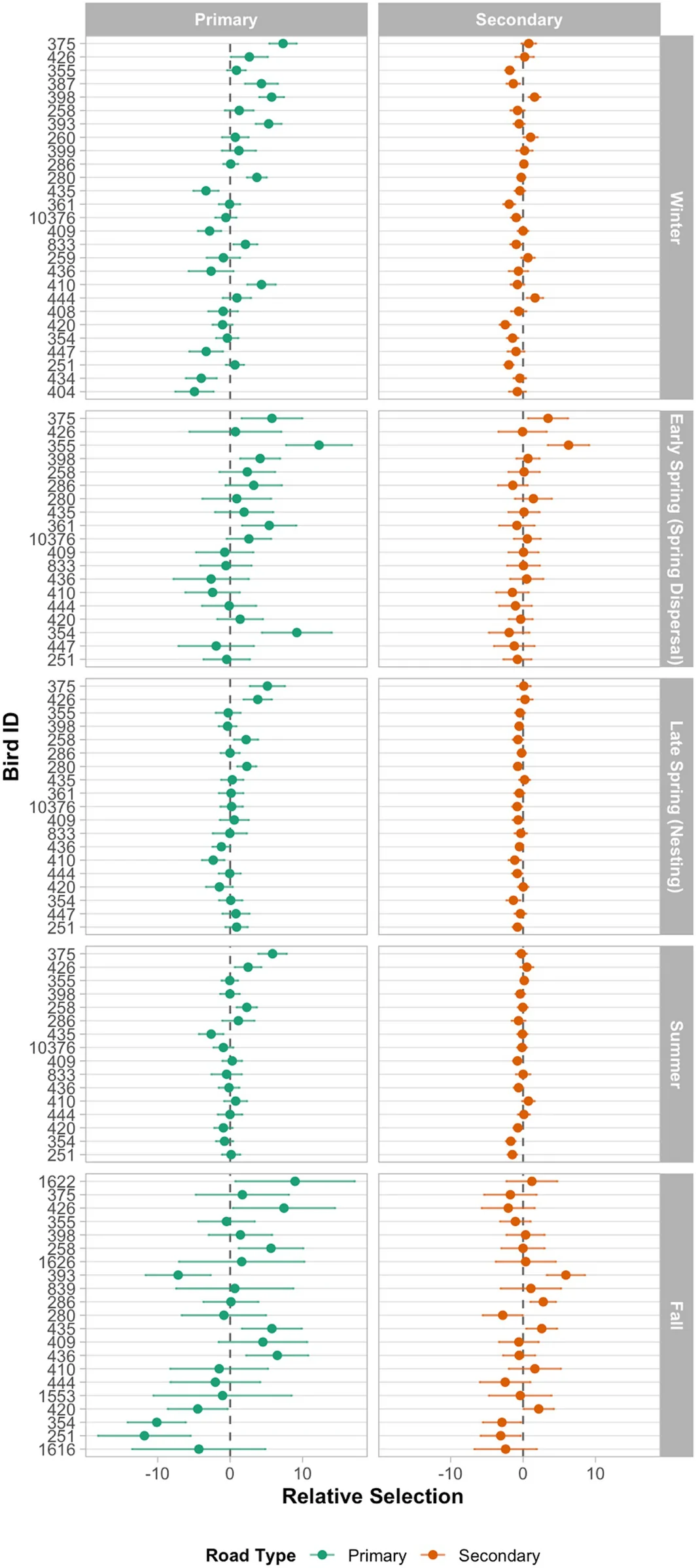
Estimates and associated 85% confidence intervals for mixed effects conditional Poisson regression fit separately for each combination of season modeling female eastern wild turkey ( *Meleagris gallopavo silvestris* ) road selection. Positive coefficients reflect selection for greater distances from roads, while negative coefficients represent selection for areas closer to roads. Habitat selection was modeled separately for winter (*n* = 27 females), early spring (*n* = 19), late spring (*n* = 19), summer (*n* = 16), and fall (*n* = 21). Females were monitored using GPS transmitters in central Maine, USA, from 2018 to 2020.

### 3.2 Diurnal Differences in Road Selection Across Seasons

During the morning hours, females selected locations closer to secondary roads in winter (−0.69; CI = −1.29 to −0.10) and summer (−1.05; CI = −1.50 to −0.59; Figure 4). At midday, females selected areas closer to secondary roads during winter (−0.67; CI = −1.11 to −0.23) and late spring (−0.11; CI = −2.02 to −0.31), but areas farther from primary roads in early spring (2.94; CI = 0.60–5.27; Figure 4). In the afternoon hours during early spring, females selected locations farther from primary roads (2.71; CI = 0.39–5.03; Figure 4).

**FIGURE 4:**
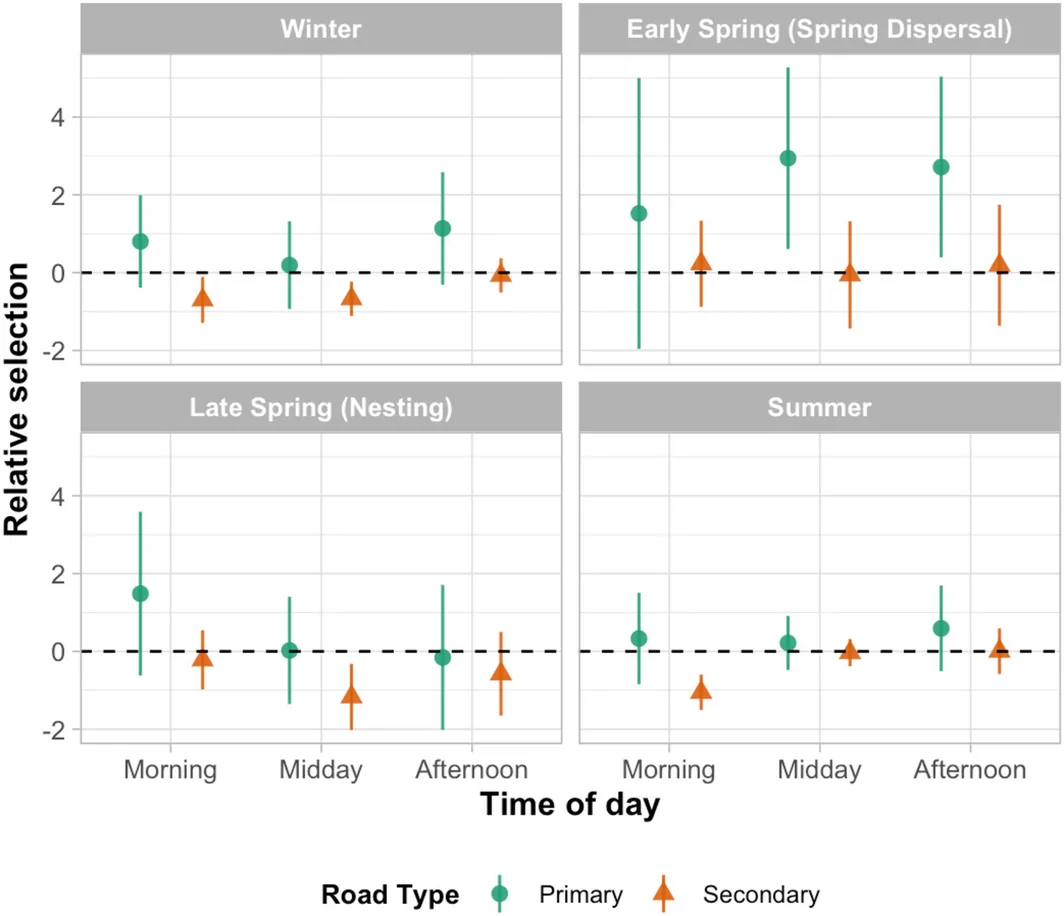
Coefficients and associated 85% confidence intervals for mixed effects conditional Poisson regression fit separately for each combination of season and time‐of‐day for modeling female eastern wild turkey ( *Meleagris gallopavo silvestris* ) road selection. Positive coefficients reflect selection for greater distances from roads, while negative coefficients represent selection for areas closer to roads. Habitat selection was modeled separately for winter (*n* = 27 females), early spring (*n* = 19), late spring (*n* = 19), summer (*n* = 16), and fall (*n* = 21). Females were monitored using GPS transmitters in central Maine, USA, from 2018 to 2020. We did not fit models for the fall season for each time of day due to a small sample size of observations in midday and afternoon hours.

### 3.3 Effect of Land Cover on Road Selection Across Seasons

We observed that females generally selected locations farther from primary and secondary roads in developed areas (Figure 5). During the winter months, relative to hardwood forest, females selected areas closer to primary roads in conifer forest cover (−0.09; CI = −0.14 to −0.05; Figure 5). In late spring, females selected areas closer to secondary roads, while selecting areas farther from secondary roads in developed areas relative to hardwood forest (Figure 5). During the summer months, relative to hardwood forest, females selected locations farther from primary (0.39; 0.31–0.46) and secondary roads (0.22; 0.13–0.32; Figure 5) in agricultural landscapes. In addition, females selected locations farther from secondary roads in developed landscapes (0.50; CI = 0.44–0.56).

**FIGURE 5:**
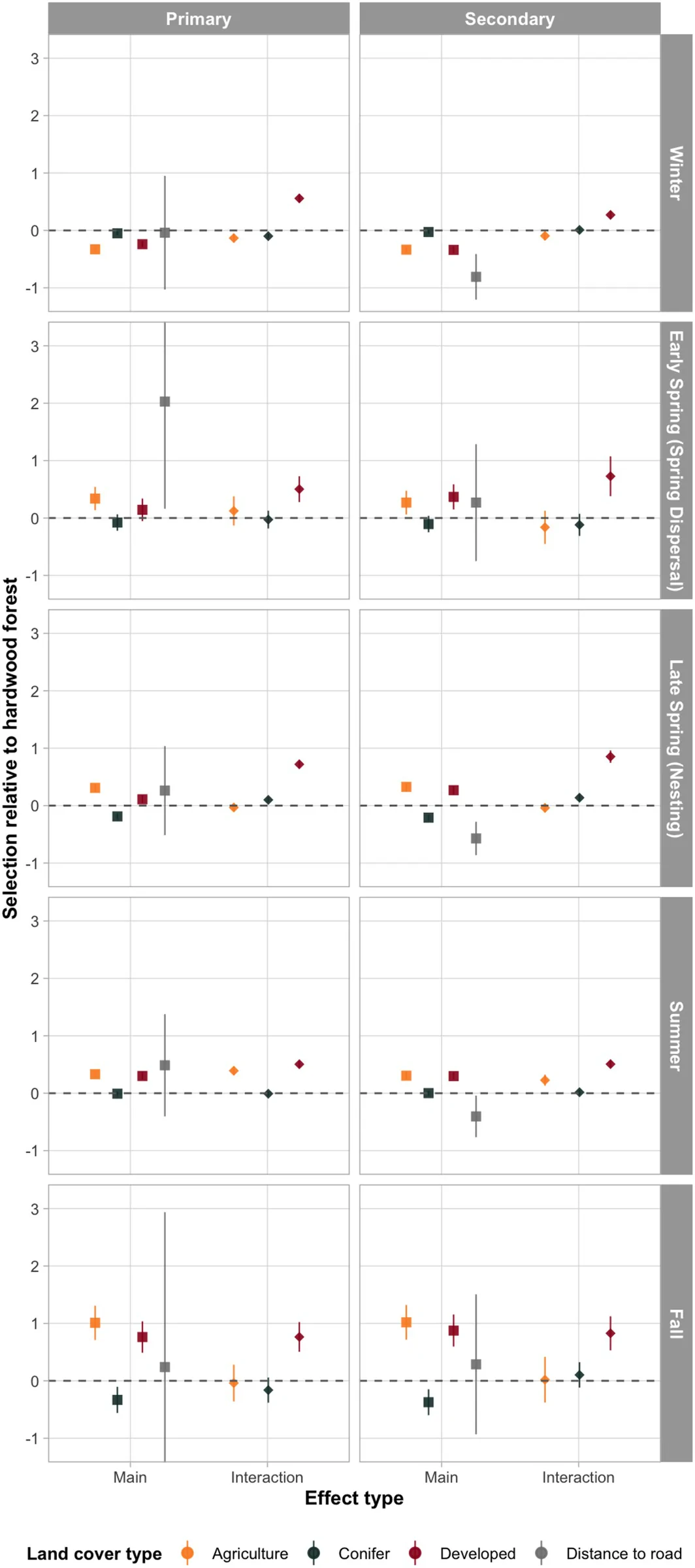
Main effect and interaction‐effect coefficients presented with associated 85% confidence intervals for mixed effects conditional Poisson regression fit separately for each season demonstrating patterns of primary and secondary road selection by female eastern wild turkeys ( *Meleagris gallopavo silvestris* ). Main effects included individual land cover variables and distance to road type, and interaction effects represented land cover variables × distance to road type. Coefficient values greater than zero indicated selection for greater distances from secondary roads, whereas values less than zero represented selection for areas closer to secondary roads; the gray dashed line at zero indicated neutral selection. Habitat selection was modeled separately for winter (*n* = 27 females), early spring (*n* = 19), late spring (*n* = 19), summer (*n* = 16), and fall (*n* = 21). Females were monitored using GPS transmitters in central Maine, USA, from 2018 to 2020.

## 4 Discussion

We investigated how turkey seasonal selection of roads was influenced by a variety of factors including road type, time‐of‐day, and landscape context. We observed general patterns such as selection for areas closer to secondary roads across many seasons and limited effects of primary roads on selection aside from early spring, where females selected areas farther from primary roads during dispersal movements. In addition, we observed selection for areas closer to secondary roads during winter and summer mornings, as well as during the midday hours in late spring, while urban development negatively influenced selection of areas near both road types during most seasons. These patterns may suggest that seasonal road selection by females is context‐dependent, varying by road type, time‐of‐day, and the surrounding landscape. For example, selection for areas near secondary roads during the morning hours may reflect movements to foraging sites as birds travel from their roost, whereas selection for areas farther from primary roads may reflect responses to increased anthropogenic disturbance. Similarly, differences in selection among land cover types suggest that roads within conifer forest cover provide either resources or cover that differs from developed and agricultural areas. Notably, we observed considerable individual variation in seasonal selection of primary and secondary roads, demonstrating heterogeneity in road selection patterns between individuals within the study. Collectively, understanding how selection contexts overlap is important as they identify periods and locations where turkey movements may coincide with human activity and roadway use with implications for vehicle collisions and management in urban areas.

The finding that females exhibited considerable individual variation in selection patterns of primary and secondary roads suggests that seasonal road selection behavior was highly variable among individuals. Rather than following a uniform pattern, some females selected areas near roads or farther from roads, with some exhibiting behavioral plasticity in selection across seasons. We also found that individual variability was considerably greater for more heavily‐traveled primary roads, which could suggest a mechanism where individuals vary in sensitivity to characteristics of vehicle travel (e.g., noise). We also observed lower individual variability during late spring and summer; it is possible that other habitat factors, such as high‐quality foraging areas during brood‐rearing play a greater role during this time of year and prevent individual flexibility in road selection. Accounting for individual variation in road selection patterns was important for this study, portraying that individual habitat selection patterns may be overlooked in models that solely focus on population‐level effects, which has been found in other studies (Gillingham and Parker 2008; Mitchell et al. 2020; Muff et al. 2020; Lesmerises and St‐Laurent 2017). For example, Gillingham and Parker (2008) found in seasonal habitat selection of moose ( *Alces alces* ) that their global model for habitat selection exhibited uniform patterns, but when accounting for individual variation, no individuals within the study exhibited consistent selection patterns. Similarly, Lesmerises and St‐Laurent (2017) found that black bears ( *Ursus americanus* ) exhibited substantial individual differences in habitat selection patterns, and that relying solely on population‐level effects may obscure these differences.

We observed seasonally variable patterns in road selection, suggesting that responses to roads were driven by changes in human activity and shifting resource requirements across the annual cycle. Females generally selected areas closer to secondary roads across many seasons with limited effects of primary roads on selection. However, an exception was during early spring dispersal movements, where individuals shifted selection patterns to areas farther from primary roads, suggesting that higher traveled state roads and highways may influence these movements. Previous wildlife studies have demonstrated roads influencing long‐distance movement behavior. For example, Passoni et al. (2021) found that both migrating and dispersing roe deer ( *Cervus elaphus* ) strongly avoided road crossings, suggesting that roads may constrain movement and functional connectivity across multiple scales. Similarly, Seidler et al. (2015) found that highways with large volumes of traffic acted as complete barriers for migrating pronghorn ( *Antilocapra americana* ) passage. Notably, we failed to detect any effects of primary roads on road selection during other seasons, suggesting that state roads and highways may influence habitat selection during spring dispersal movements, but not during other periods of the annual cycle where individuals may be more stationary. However, our GPS dataset contained a small sample size of locations during spring dispersal movements (*n* = 949) so more research investigating the influence of roads on female spring dispersal behavior may be needed.

Diurnal variation in road selection suggests that females adjusted their space use patterns in response to trade‐offs between foraging opportunities and disturbance. Previous studies have documented wildlife altering diel selection patterns to mitigate anthropogenic interactions. For example, Borowik et al. (2024) found that moose ( *Alces alces* ) avoided roads during daylight hours and selected locations closer to roads in the evening, likely due to forage opportunities along roadsides and reduced risk of anthropogenic disturbance. Similarly, Meisingset et al. (2012) found that red deer avoided roads during the day and crossed roads near optimal foraging habitat. In this study, females selected areas closer to secondary roads during morning hours in winter and summer, likely reflecting the use of lower traveled roads to provide pathways to foraging opportunities. Morning hours are associated with increased foraging behavior off the roost, and plowed secondary roads may promote ease of travel by lowering energetic costs during the winter months (Barnum 2003; Thompson and Henderson 1998) while providing early successional vegetation and exposed substrates that enhance foraging efficiency during the summer (Lambert 1986; Butler et al. 2005; Delahunt 2011). However, we failed to detect this pattern with primary roads during the morning hours, suggesting that greater traffic volumes and anthropogenic disturbance may potentially outweigh foraging benefits.

Our findings of selection for areas closer to secondary roads in summer mornings, may largely reflect seasonal differences in female behavior and thermoregulatory benefits. For example, during summer mornings, females with broods tend to be more mobile, capitalizing on cooler morning temperatures to exploit foraging opportunities as offspring (i.e., poults) are sensitive to their thermal environment (Roberts and Porter 1998; Rakowski et al. 2019). During brood‐rearing, female movements are specifically linked to early successional areas as these locations provide nutrient rich forage that aid in poult growth and development (Chamberlain et al. 2020; Nelson et al. 2022, 2023). Females sometimes make recursive movements to capitalize on optimal foraging patches with poults often revisiting favored early successional patches multiple times (Bakner et al. 2022, 2023). While we were unable to quantify fine‐scale vegetative attributes (e.g., grasses/forbs) and anthropogenic mowing along road edges, land cover composition within 50 m of primary and secondary roads was largely dominated by developed land use within the study area where mowing is likely occurring (Table S7).

We observed differential patterns of turkey selection of roads when accounting for the effect of the surrounding landscape of roads across seasons. Our findings aligned with previous studies suggesting that landscape context is an important predictor in road selection, with turkeys previously shown to avoid high traffic roads (McDougal et al. 1990; Gerrits 2019; Carl et al. 2023) and roads in urban areas specifically (Collins et al. 2025). Similarly, we observed that females generally selected locations farther from primary and secondary roads in developed areas. Turkeys have highly developed hearing and precise eyesight (Healy and Dickson 1992) and may not be able to communicate effectively or visualize the landscape in areas with high traffic volumes. In contrast, females selected areas closer to primary roads when those roads occurred within conifer forest cover during the winter months. Turkeys have been documented restricting space use and shifting to a stationary behavioral state in conifer forest cover during harsh winter weather conditions (Vander Haegen et al. 1989; Niedzielski and Bowman 2016; Gonnerman et al. 2023), likely because conifer forest cover provides thermoregulatory benefits and reduced snow accumulation (Ozoga and Gysel 1972; Nelson 1995; Lishawa et al. 2007; DelGiudice et al. 2013). Consequently, females may have tolerated closer proximity to primary roads in these areas because turkeys in Maine also persist near their northern range limit, where thermoregulation during winter months is likely a primary ecological constraint. Such behavior would be consistent with the general observation that wintering turkeys display dynamic behaviors in response to changing weather conditions (e.g., cold and snow; Gonnerman et al. 2023).

Our results demonstrate that road ecology may play an important role in shaping seasonal habitat selection patterns of females with implications for urban wildlife management, tourism, and vehicle collision risk. By evaluating seasonal habitat selection patterns as a function of road type, time‐of‐day, and surrounding landscape, we found that female selection of roads was context‐dependent. Specifically, our findings demonstrate that individuals may weigh tradeoffs in seasonal habitat selection with a variety of factors including perceived threat of predation, resource requirements, and thermoregulatory challenges. Our results also provide context for species management, as females selected areas closer to primary and secondary roads during various seasons, times of day, and land cover contexts. Specifically, females selected locations closer to secondary roads during summer mornings, and primary roads within areas comprised of conifer forest cover during the winter months, which has implications for work commutes and tourism throughout the state. Our findings have implications for understanding the temporal risk of wildlife‐vehicle collisions and suggest that factors such as season, time‐of‐day, and the surrounding landscape context of roads influence habitat selection, warranting consideration when evaluating the ecological effects of roads.

## Author Contributions

**Kyle J. Smelter:** conceptualization (equal), data curation (supporting), formal analysis (equal), investigation (equal), methodology (equal), validation (equal), visualization (equal), writing – original draft (equal), writing – review and editing (equal). **Erik J. Blomberg:** conceptualization (equal), formal analysis (equal), funding acquisition (equal), investigation (equal), methodology (equal), project administration (equal), supervision (equal), validation (equal), visualization (equal), writing – original draft (equal), writing – review and editing (equal). **Liam A. Berigan:** formal analysis (equal), investigation (equal), methodology (equal), visualization (equal), writing – original draft (equal), writing – review and editing (equal). **Matthew B. Gonnerman:** conceptualization (equal), data curation (lead), formal analysis (equal), investigation (equal), methodology (equal), resources (equal), validation (equal), visualization (equal), writing – original draft (equal), writing – review and editing (equal). **Stephanie A. Shea:** investigation (equal), methodology (equal), resources (equal), writing – review and editing (equal). **Veronica A. Winter:** investigation (equal), methodology (equal), writing – review and editing (equal). **Kelsey Sullivan:** funding acquisition (equal), investigation (equal), methodology (equal), project administration (equal), resources (equal). **Pauline L. Kamath:** funding acquisition (equal), investigation (equal), methodology (equal), project administration (equal), writing – review and editing (equal). **Frances E. Buderman:** formal analysis (equal), investigation (equal), methodology (equal), supervision (equal), visualization (equal), writing – review and editing (equal).

## Funding

This project was funded by the Maine Department of Inland Fisheries and Wildlife, the National Wild Turkey Federation, the Maine State Chapter of the National Wild Turkey Federation, the Maine Outdoor Heritage Fund (Award No. 182‐01‐08), the University of Maine Graduate Student Government, and the USDA‐National Institute of Food and Agriculture Hatch Project Number ME0‐21908 and McIntire‐Stennis Project Number ME0‐41602 through the Maine Agricultural and Forest Experiment Station.

## Ethics Statement

All animal handling protocols were approved by the University of Maine's Institutional Animal Care and Use Committee (IACUC Protocol # A2017_11_03).

## Conflicts of Interest

The authors declare no conflicts of interest.

## Supporting information


**Figure S1:** Estimates and associated 95% confidence intervals for mixed effects conditional Poisson regression fit separately for each combination of season modeling female eastern wild turkey ( *Meleagris gallopavo silvestris* ) road selection. Positive coefficients reflect selection for greater distances from roads, while negative coefficients represent selection for areas closer to roads. Habitat selection was modeled separately for winter (*n* = 27 females), early spring (*n* = 19), late spring (*n* = 19), summer (*n* = 16), and fall (*n* = 21). Females were monitored using GPS transmitters in central Maine, USA, from 2018 to 2020.
**Figure S2:** Coefficients and associated 95% confidence intervals for mixed effects conditional Poisson regression fit separately for each combination of season and time‐of‐day for modeling female eastern wild turkey ( *Meleagris gallopavo silvestris* ) road selection. Positive coefficients reflect selection for greater distances from roads, while negative coefficients represent selection for areas closer to roads. Habitat selection was modeled separately for winter (*n* = 27 females), early spring (*n* = 19), late spring (*n* = 19), summer (*n* = 16), and fall (*n* = 21). Females were monitored using GPS transmitters in central Maine, USA, from 2018 to 2020. We did not fit models for the fall season for each time of day due to a small sample size of observations in midday and afternoon hours.
**Figure S3:** Main effect and interaction‐effect coefficients presented with associated 95% confidence intervals for mixed effects conditional Poisson regression fit separately for each season demonstrating patterns of primary and secondary road selection by female eastern wild turkeys ( *Meleagris gallopavo silvestris* ). Main effects included individual land cover variables and distance to road type, and interaction effects represented land cover variables × distance to road type. Coefficient values greater than zero indicated selection for greater distances from secondary roads, whereas values less than zero represented selection for areas closer to secondary roads; the gray dashed line at zero indicated neutral selection. Habitat selection was modeled separately for winter (*n* = 27 females), early spring (*n* = 19), late spring (*n* = 19), summer (*n* = 16), and fall (*n* = 21). Females were monitored using GPS transmitters in central Maine, USA, from 2018 to 2020.
**Table S1:** Variance components for individual random slopes when modeling seasonal road selection patterns (Objective 1) for female eastern wild turkeys ( *Meleagris gallopavo silvestris* ) in Central Maine USA (2018–2020). Estimates were derived from a mixed‐effects conditional logistic regression with a random intercept for stratum ID and an individual random slope, with the latter accounting for individual variation in road selection patterns.
**Table S2:** Variance components for individual random slopes when modeling time‐of‐day differences in seasonal road selection patterns (Objective 2) for female eastern wild turkeys ( *Meleagris gallopavo silvestris* ) in Central Maine USA (2018–2020). Estimates were derived from a mixed‐effects conditional logistic regression with a random intercept for stratum ID and an individual random slope, with the latter accounting for individual variation in road selection patterns.
**Table S3:** Variance components for individual random slopes when modeling differences in seasonal road selection patterns as a function of land cover type (Objective 3) for female eastern wild turkeys ( *Meleagris gallopavo silvestris* ) in Central Maine USA (2018–2020). Estimates were derived from a mixed‐effects conditional logistic regression with a random intercept for stratum ID and an individual random slope, with the latter accounting for individual variation in road selection patterns.
**Table S4:** Estimates and associated 85% confidence intervals for mixed effects conditional Poisson regression fit separately for each combination of season modeling female eastern wild turkey ( *Meleagris gallopavo silvestris* ) road selection. Results are presented in the context of relative selection strength, where coefficient values above one reflect selection for greater distances from roads, while coefficients below one represent selection for areas closer to roads. Habitat selection was modeled separately for winter (*n* = 27 females), early spring (*n* = 19), late spring (*n* = 19), summer (*n* = 16), and fall (*n* = 21). Females were monitored using GPS transmitters in central Maine, USA, from 2018 to 2020.
**Table S5:** Estimates and associated 85% confidence intervals for mixed effects conditional Poisson regression fit separately for each combination of season and time‐of‐day modeling female eastern wild turkey ( *Meleagris gallopavo silvestris* ) road selection. Results are presented in the context of relative selection strength, where coefficient values above one reflect selection for greater distances from roads, while coefficients below one represent selection for areas closer to roads. Habitat selection was modeled separately for winter (*n* = 27 females), early spring (*n* = 19), late spring (*n* = 19), summer (*n* = 16), and fall (*n* = 21). Females were monitored using GPS transmitters in central Maine, USA, from 2018 to 2020.
**Table S6:** Estimates and associated 85% confidence intervals for mixed effects conditional Poisson regression fit separately for each combination of season modeling female eastern wild turkey ( *Meleagris gallopavo silvestris* ) road selection as a function of land cover type. Results are presented in the context of relative selection strength, where coefficient values above one reflect selection for greater distances from roads, while coefficients below one represent selection for areas closer to roads. Habitat selection was modeled separately for winter (*n* = 27 females), early spring (*n* = 19), late spring (*n* = 19), summer (*n* = 16), and fall (*n* = 21). Females were monitored using GPS transmitters in central Maine, USA, from 2018 to 2020.
**Table S7:** Land cover composition within 50 m of primary and secondary roads available to female eastern wild turkeys ( *Meleagris gallopavo silvestris* ) in central Maine, USA (2018–2020). Values represent the proportion of raster cells in each land cover class within 50 m of each road type cropped to the study area, with total cell counts shown in parentheses.

## Data Availability

The data and R code are available in a Dryad repository DOI: https://doi.org/10.5061/dryad.zgmsbcctf.
