# Supplementary material for: Female Wild Turkeys Exhibit Context Dependence in Their Selection of Roads
Source: Ecol Evol. 2026 Oct 14;16(10):e74468. doi: 10.1002/ece3.74468 (PMC13668704; doi:10.1002/ece3.74468)
Supplement: Supplementary file 1 [file ECE3-16-e74468-s001.docx]

**APPENDIX: Supplemental Files**

**JOURNAL NAME:** Ecology and Evolution

**MANUSCRIPT TITLE**: Female Wild Turkeys Exhibit Context Dependence in Their Selection of Roads

Supplementary Figure S1. Estimates and associated 95% confidence intervals for mixed effects conditional Poisson regression fit separately for each combination of season modeling female eastern wild turkey (*Meleagris gallopavo silvestris*) road selection. Positive coefficients reflect selection for greater distances from roads, while negative coefficients represent selection for areas closer to roads. Habitat selection was modeled separately for winter (n = 27 females), early spring (n = 19), late spring (n = 19), summer (n = 16), and fall (n = 21). Females were monitored using GPS transmitters in central Maine, USA, from 2018–2020.

Supplementary Figure S2. Coefficients and associated 95% confidence intervals for mixed effects conditional Poisson regression fit separately for each combination of season and time-of-day for modeling female eastern wild turkey (*Meleagris gallopavo silvestris*) road selection. Positive coefficients reflect selection for greater distances from roads, while negative coefficients represent selection for areas closer to roads. Habitat selection was modeled separately for winter (n = 27 females), early spring (n = 19), late spring (n = 19), summer (n = 16), and fall (n = 21). Females were monitored using GPS transmitters in central Maine, USA, from 2018–2020. We did not fit models for the fall season for each time of day due to a small sample size of observations in midday and afternoon hours.

Supplementary Figure S3. Main effect and interaction-effect coefficients presented with associated 95% confidence intervals for mixed effects conditional Poisson regression fit separately for each season demonstrating patterns of primary and secondary road selection by female eastern wild turkeys (*Meleagris gallopavo silvestris*). Main effects included individual land cover variables and distance to road type, and interaction effects represented land cover variables × distance to road type. Coefficient values greater than zero indicated selection for greater distances from secondary roads, whereas values less than zero represented selection for areas closer to secondary roads; the gray dashed line at zero indicated neutral selection. Habitat selection was modeled separately for winter (n = 27 females), early spring (n = 19), late spring (n = 19), summer (n = 16), and fall (n = 21). Females were monitored using GPS transmitters in central Maine, USA, from 2018–2020.

Supplementary Table S1. Variance components for individual random slopes when modeling seasonal road selection patterns (Objective 1) for female eastern wild turkeys (*Meleagris gallopavo silvestris*) in Central Maine USA (2018–2020). Estimates were derived from a mixed-effects conditional logistic regression with a random intercept for stratum ID and an individual random slope, with the latter accounting for individual variation in road selection patterns.

| Model | Estimated  Individual Variance |
| --- | --- |
| Win.Prim | 10.87 |
| Win.Sec | 1.40 |
| Spr.Prim | 21.71 |
| Spr.Sec | 5.76 |
| Nest.Prim | 4.12 |
| Nest.Sec | 0.38 |
| Sum.Prim | 4.32 |
| Sum.Sec | 0.61 |
| Fall.Prim | 46.64 |
| Fall.Sec | 9.43 |

Abbreviations: Win. = Winter, Spr. = Spring Dispersal, Nest. = Nesting, Sum. = Summer, Prim. = Primary Road, Sec. = Secondary Road

Supplementary Table S2. Variance components for individual random slopes when modeling time-of-day differences in seasonal road selection patterns (Objective 2) for female eastern wild turkeys (*Meleagris gallopavo silvestris*) in Central Maine USA (2018–2020). Estimates were derived from a mixed-effects conditional logistic regression with a random intercept for stratum ID and an individual random slope, with the latter accounting for individual variation in road selection patterns.

| Model | Estimated  Individual Variance |
| --- | --- |
| Win.Prim.Mor | 13.83 |
| Win.Prim.Mid | 12.39 |
| Win.Prim.Aft | 21.34 |
| Win.Sec.Mor | 3.09 |
| Win.Sec.Mid | 1.39 |
| Win.Sec.Aft | 0.98 |
| Spr.Prim.Mor | 77.26 |
| Spr.Prim.Mid | 22.37 |
| Spr.Prim.Aft | 17.47 |
| Spr.Sec.Mor | 2.72 |
| Spr.Sec.Mid | 6.20 |
| Spr.Sec.Aft | 11.02 |
| Nest.Prim.Mor | 36.36 |
| Nest.Prim.Mid | 11.97 |
| Nest.Prim.Aft | 26.41 |
| Nest.Sec.Mor | 4.0 |
| Nest.Sec.Mid | 4.70 |
| Nest.Sec.Aft | 8.76 |
| Sum.Prim.Mor | 7.45 |
| Sum.Prim.Mid | 0.50 |
| Sum.Prim.Aft | 6.05 |
| Sum.Sec.Mor | 0.65 |
| Sum.Sec.Mid | <0.01 |
| Sum.Sec.Aft | 1.58 |

Abbreviations: Win. = Winter, Spr. = Spring Dispersal, Nest. = Nesting, Sum. = Summer, Prim. = Primary Road, Sec. = Secondary Road, Mor. = Morning, Mid. = Midday, Aft. = Afternoon

Supplementary Table S3. Variance components for individual random slopes when modeling differences in seasonal road selection patterns as a function of land cover type (Objective 3) for female eastern wild turkeys (*Meleagris gallopavo silvestris*) in Central Maine USA (2018–2020). Estimates were derived from a mixed-effects conditional logistic regression with a random intercept for stratum ID and an individual random slope, with the latter accounting for individual variation in road selection patterns.

| Model | Estimated  Individual Variance |
| --- | --- |
| Win.Prim | 10.95 |
| Win.Sec | 1.53 |
| Spr.Prim | 20.70 |
| Spr.Sec | 5.29 |
| Nest.Prim | 3.76 |
| Nest.Sec | 0.27 |
| Sum.Prim | 4.75 |
| Sum.Sec | 0.60 |
| Fall.Prim | 45.83 |
| Fall.Sec | 6.45 |

Abbreviations: Win. = Winter, Spr. = Spring Dispersal, Nest. = Nesting, Sum. = Summer, Prim. = Primary Road, Sec. = Secondary Road

Supplementary Table S4. Estimates and associated 85% confidence intervals for mixed effects conditional Poisson regression fit separately for each combination of season modeling female eastern wild turkey (*Meleagris gallopavo silvestris*) road selection. Results are presented in the context of relative selection strength, where coefficient values above one reflect selection for greater distances from roads, while coefficients below one represent selection for areas closer to roads. Habitat selection was modeled separately for winter (n = 27 females), early spring (n = 19), late spring (n = 19), summer (n = 16), and fall (n = 21). Females were monitored using GPS transmitters in central Maine, USA, from 2018–2020.

| Season | Road Type | Estimate | Lower  85% CI | Upper  85% CI |
| --- | --- | --- | --- | --- |
| Winter | Primary | 1.80 | 0.67 | 0.81 |
| Winter | Secondary | 0.61 | 0.42 | 0.90 |
| Spring Dispersal | Primary | 8.66 | 1.31 | 56.94 |
| Spring Dispersal | Secondary | 1.22 | 0.43 | 3.40 |
| Nesting | Primary | 1.75 | 0.79 | 3.88 |
| Nesting | Secondary | 0.61 | 0.45 | 0.83 |
| Summer | Primary | 1.54 | 0.66 | 3.60 |
| Summer | Secondary | 0.71 | 0.49 | 1.02 |
| Fall | Primary | 0.98 | 0.06 | 14.29 |
| Fall | Secondary | 0.91 | 0.24 | 3.47 |

Supplementary Table S5. Estimates and associated 85% confidence intervals for mixed effects conditional Poisson regression fit separately for each combination of season and time-of-day modeling female eastern wild turkey (*Meleagris gallopavo silvestris*) road selection. Results are presented in the context of relative selection strength, where coefficient values above one reflect selection for greater distances from roads, while coefficients below one represent selection for areas closer to roads. Habitat selection was modeled separately for winter (n = 27 females), early spring (n = 19), late spring (n = 19), summer (n = 16), and fall (n = 21). Females were monitored using GPS transmitters in central Maine, USA, from 2018–2020.

| Season | Road Type | Time | Estimate | Lower  85% CI | Upper  85% CI |
| --- | --- | --- | --- | --- | --- |
| Winter | Primary | Morning | 2.23 | 0.67 | 7.32 |
| Winter | Primary | Midday | 1.21 | 0.31 | 3.74 |
| Winter | Primary | Afternoon | 3.11 | 0.73 | 13.23 |
| Winter | Secondary | Morning | 0.49 | 0.27 | 0.89 |
| Winter | Secondary | Midday | 0.51 | 0.32 | 0.79 |
| Winter | Secondary | Afternoon | 0.93 | 0.60 | 1.44 |
| Spring Dispersal | Primary | Morning | 4.58 | 0.14 | 149.12 |
| Spring Dispersal | Primary | Midday | 18.95 | 1.83 | 195.82 |
| Spring  Dispersal | Primary | Afternoon | 15.10 | 1.48 | 154.03 |
| Spring  Dispersal | Secondary | Morning | 1.25 | 0.41 | 3.81 |
| Spring Dispersal | Secondary | Midday | 0.94 | 0.23 | 3.75 |
| Spring Dispersal | Secondary | Afternoon | 1.21 | 0.25 | 5.73 |
| Nesting | Primary | Morning | 4.41 | 0.53 | 36.24 |
| Nesting | Primary | Midday | 1.02 | 0.25 | 4.06 |
| Nesting | Primary | Afternoon | 0.85 | 0.13 | 5.52 |
| Nesting | Secondary | Morning | 0.80 | 0.37 | 1.71 |
| Nesting | Secondary | Midday | 0.31 | 0.25 | 0.72 |
| Nesting | Secondary | Afternoon | 0.56 | 0.19 | 1.64 |
| Summer | Primary | Morning | 1.39 | 0.43 | 4.50 |
| Summer | Primary | Midday | 1.24 | 0.62 | 2.48 |
| Summer | Primary | Afternoon | 1.80 | 0.60 | 5.42 |
| Summer | Secondary | Morning | 0.34 | 0.22 | 0.55 |
| Summer | Secondary | Midday | 0.96 | 0.68 | 1.37 |
| Summer | Secondary | Afternoon | 1.00 | 0.56 | 1.80 |

Supplementary Table S6. Estimates and associated 85% confidence intervals for mixed effects conditional Poisson regression fit separately for each combination of season modeling female eastern wild turkey (*Meleagris gallopavo silvestris*) road selection as a function of land cover type. Results are presented in the context of relative selection strength, where coefficient values above one reflect selection for greater distances from roads, while coefficients below one represent selection for areas closer to roads. Habitat selection was modeled separately for winter (n = 27 females), early spring (n = 19), late spring (n = 19), summer (n = 16), and fall (n = 21). Females were monitored using GPS transmitters in central Maine, USA, from 2018–2020.

| Season | Road Type | Variable | Effect | Estimate | Lower  85% CI | Upper  85% CI |
| --- | --- | --- | --- | --- | --- | --- |
| Winter | Primary | Agriculture | Main | 0.72 | 0.68 | 0.76 |
| Winter | Primary | Conifer | Main | 0.95 | 0.92 | 0.98 |
| Winter | Primary | Developed | Main | 0.79 | 0.75 | 0.83 |
| Winter | Primary | Distance to Primary Road | Main | 0.96 | 0.36 | 2.59 |
| Winter | Primary | Distance to Primary Road × Agriculture | Interaction | 0.87 | 0.81 | 0.94 |
| Winter | Primary | Distance to Primary Road × Conifer | Interaction | 0.90 | 0.87 | 0.94 |
| Winter | Primary | Distance to Primary Road × Developed | Interaction | 1.75 | 1.63 | 1.88 |
| Winter | Secondary | Agriculture | Main | 8.66 | 1.31 | 56.94 |
| Winter | Secondary | Conifer | Main | 1.22 | 0.43 | 3.40 |
| Winter | Secondary | Developed | Main | 1.75 | 0.79 | 3.88 |
| Winter | Secondary | Distance to Secondary Road | Main | 0.45 | 0.30 | 0.66 |
| Winter | Secondary | Distance to Secondary Road × Agriculture | Interaction | 0.91 | 0.84 | 0.98 |
| Winter | Secondary | Distance to Secondary Road × Conifer | Interaction | 1.01 | 0.98 | 1.04 |
| Winter | Secondary | Distance to Secondary Road × Developed | Interaction | 1.31 | 1.23 | 1.40 |
| Spring Dispersal | Primary | Agriculture | Main | 1.40 | 1.15 | 1.72 |
| Spring Dispersal | Primary | Conifer | Main | 0.92 | 0.80 | 1.07 |
| Spring Dispersal | Primary | Developed | Main | 1.15 | 0.95 | 1.40 |
| Spring Dispersal | Primary | Distance to Primary Road | Main | 7.60 | 1.18 | 49.11 |
| Spring Dispersal | Primary | Distance to Primary Road × Agriculture | Interaction | 1.13 | 0.88 | 1.46 |
| Spring Dispersal | Primary | Distance to Primary Road × Conifer | Interaction | 0.97 | 0.83 | 1.14 |
| Spring Dispersal | Primary | Distance to Primary Road × Developed | Interaction | 1.65 | 1.32 | 2.07 |
| Spring Dispersal | Secondary | Agriculture | Main | 1.31 | 1.06 | 1.61 |
| Spring Dispersal | Secondary | Conifer | Main | 0.90 | 0.78 | 1.04 |
| Spring Dispersal | Secondary | Developed | Main | 1.44 | 1.16 | 1.80 |
| Spring Dispersal | Secondary | Distance to Secondary Road | Main | 1.31 | 1.47 | 3.62 |
| Spring Dispersal | Secondary | Distance to Secondary Road × Agriculture | Interaction | 0.85 | 0.64 | 1.13 |
| Spring Dispersal | Secondary | Distance to Secondary Road × Conifer | Interaction | 0.89 | 0.73 | 1.08 |
| Spring Dispersal | Secondary | Distance to Primary Road × Developed | Interaction | 2.07 | 1.46 | 2.93 |
| Nesting | Primary | Agriculture | Main | 1.36 | 1.28 | 1.46 |
| Nesting | Primary | Conifer | Main | 0.83 | 0.78 | 0.87 |
| Nesting | Primary | Developed | Main | 1.12 | 1.04 | 1.20 |
| Nesting | Primary | Distance to Primary Road | Main | 1.30 | 0.60 | 2.82 |
| Nesting | Primary | Distance to Primary Road × Agriculture | Interaction | 0.97 | 0.90 | 1.04 |
| Nesting | Primary | Distance to Primary Road × Conifer | Interaction | 1.11 | 1.06 | 1.15 |
| Nesting | Primary | Distance to Primary Road × Developed | Interaction | 2.05 | 1.92 | 2.19 |
| Nesting | Secondary | Agriculture | Main | 1.39 | 1.30 | 1.48 |
| Nesting | Secondary | Conifer | Main | 0.81 | 0.77 | 0.86 |
| Nesting | Secondary | Developed | Main | 1.31 | 1.22 | 1.40 |
| Nesting | Secondary | Distance to Secondary Road | Main | 0.56 | 0.42 | 0.76 |
| Nesting | Secondary | Distance to Secondary Road × Agriculture | Interaction | 0.96 | 0.89 | 1.04 |
| Nesting | Secondary | Distance to Secondary Road × Conifer | Interaction | 1.15 | 1.08 | 1.22 |
| Nesting | Secondary | Distance to Secondary Road × Developed | Interaction | 2.35 | 2.11 | 2.62 |
| Summer | Primary | Agriculture | Main | 1.39 | 1.31 | 1.48 |
| Summer | Primary | Conifer | Main | 0.99 | 0.95 | 1.04 |
| Summer | Primary | Developed | Main | 1.35 | 1.27 | 1.44 |
| Summer | Primary | Distance to Primary Road | Main | 1.63 | 0.67 | 3.96 |
| Summer | Primary | Distance to Primary Road × Agriculture | Interaction | 1.48 | 1.37 | 1.59 |
| Summer | Primary | Distance to Primary Road × Conifer | Interaction | 0.99 | 0.95 | 1.03 |
| Summer | Primary | Distance to Primary Road × Developed | Interaction | 1.66 | 1.56 | 1.76 |
| Summer | Secondary | Agriculture | Main | 1.36 | 1.27 | 1.45 |
| Summer | Secondary | Conifer | Main | 1.00 | 0.96 | 1.05 |
| Summer | Secondary | Developed | Main | 1.35 | 1.27 | 1.44 |
| Summer | Secondary | Distance to Secondary Road | Main | 0.67 | 0.46 | 0.96 |
| Summer | Secondary | Distance to Secondary Road × Agriculture | Interaction | 1.26 | 1.14 | 1.38 |
| Summer | Secondary | Distance to Secondary Road × Conifer | Interaction | 1.02 | 0.98 | 1.06 |
| Summer | Secondary | Distance to Secondary Road × Developed | Interaction | 1.66 | 1.57 | 1.76 |
| Fall | Primary | Agriculture | Main | 2.75 | 2.04 | 3.70 |
| Fall | Primary | Conifer | Main | 0.72 | 0.57 | 0.90 |
| Fall | Primary | Developed | Main | 2.14 | 1.63 | 2.82 |
| Fall | Primary | Distance to Primary Road | Main | 1.27 | 0.09 | 18.85 |
| Fall | Primary | Distance to Primary Road × Agriculture | Interaction | 0.96 | 0.70 | 1.32 |
| Fall | Primary | Distance to Primary Road × Conifer | Interaction | 0.85 | 0.68 | 1.06 |
| Fall | Primary | Distance to Primary Road × Developed | Interaction | 2.15 | 1.66 | 2.78 |
| Fall | Secondary | Agriculture | Main | 2.77 | 2.05 | 3.75 |
| Fall | Secondary | Conifer | Main | 0.69 | 0.55 | 0.86 |
| Fall | Secondary | Developed | Main | 2.40 | 1.82 | 3.17 |
| Fall | Secondary | Distance to Secondary Road | Main | 1.33 | 0.39 | 4.51 |
| Fall | Secondary | Distance to Secondary Road × Agriculture | Interaction | 1.26 | 1.14 | 1.38 |
| Fall | Secondary | Distance to Secondary Road × Conifer | Interaction | 1.11 | 0.89 | 1.38 |
| Fall | Secondary | Distance to Secondary Road × Developed | Interaction | 2.29 | 1.70 | 3.08 |

Supplementary Table S7. Land cover composition within 50 m of primary and secondary roads available to female eastern wild turkeys (*Meleagris gallopavo silvestris*) in central Maine, USA (2018–2020). Values represent the proportion of raster cells in each land cover class within 50 m of each road type cropped to the study area, with total cell counts shown in parentheses.

| Land Cover Type | Primary Road  ≤ 50 m | Secondary Road  ≤ 50 m |
| --- | --- | --- |
| Developed | 0.57  (254612) | 0.52  (353452) |
| Hardwood | 0.09  (41115) | 0.11  (74703) |
| Conifer | 0.23  (98016) | 0.25  (167599) |
| Agriculture | 0.07  (34248) | 0.08  (58536) |
| Other | 0.03  (14132) | 0.02  (15745) |
